# Myoendothelial Junctions of Mature Coronary Vessels Express Notch Signaling Proteins

**DOI:** 10.3389/fphys.2020.00029

**Published:** 2020-02-04

**Authors:** Patricia E. McCallinhart, Lauren A. Biwer, Olivia E. Clark, Brant E. Isakson, Brenda Lilly, Aaron J. Trask

**Affiliations:** ^1^Center for Cardiovascular Research, The Heart Center, The Research Institute at Nationwide Children’s Hospital, Columbus, OH, United States; ^2^Robert M. Berne Cardiovascular Research Center, University of Virginia School of Medicine, Charlottesville, VA, United States; ^3^Department of Molecular Physiology and Biophysics, University of Virginia School of Medicine, Charlottesville, VA, United States; ^4^Department of Pediatrics, The Ohio State University College of Medicine, Columbus, OH, United States

**Keywords:** myoendothelial junction, Notch, vascular, internal elastic lamina, coronary

## Abstract

**Rationale:**

Myoendothelial junctions (MEJs) within the fenestrae of the internal elastic lamina (IEL) are critical sites that allow for endothelial cell (EC) - vascular smooth muscle cell (VSMC) contact and communication. Vascular Notch signaling is a critical determinant of normal vasculogenesis and remodeling, and it regulates cell phenotype via contact between ECs and VSMCs. To date, no studies have linked Notch signaling to the MEJ despite it requiring cell-cell contact. Furthermore, very little is known about Notch in the adult coronary circulation or the localization of Notch signaling and activity within the mature intact blood vessel.

**Objective:**

We tested the hypothesis that vascular Notch signaling between ECs and VSMCs occurs at MEJs.

**Methods and Results:**

Notch receptor and ligand immunofluorescence was performed in human coronary EC and VSMC co-cultures across transwell inserts (*in vitro* MEJs) and in the intact mouse coronary circulation. Human coronary VSMC Notch activity induced by human coronary ECs at the *in vitro* MEJ was assessed using a CBF-luciferase construct. We observed Jagged1, Notch1, Notch2, and Notch3 expression within the *in vitro* and *in vivo* MEJs. We also demonstrated a 3-fold induction (*p* < 0.001) of human coronary VSMC Notch signaling by ECs at the *in vitro* MEJ, which was completely blocked by the Notch inhibitor, DAPT (*p* < 0.01).

**Conclusion:**

We demonstrate for the first time in mature blood vessels that Notch receptors and ligands are expressed within and are active at coronary MEJs, demonstrating a previously unrecognized mode of Notch signaling regulation between the endothelium and smooth muscle.

## Introduction

Heterocellular communication between endothelial cells (ECs) and vascular smooth muscle cells (VSMCs) is a demonstrated component to normal vascular development and physiology, ultimately dictating vascular function and vessel remodeling (Rostama et al., 2014). The internal elastic lamina (IEL) is a layer of elastin that separates ECs lining the lumen and the VSMCs that comprise the media layer of blood vessels. It is important to note that in large conduit arteries such as the aorta, there are little to no fenestrae within the IEL, limiting the direct contact and communication between the ECs and VSMCs; however, as the diameter of the vessel decreases, the number of fenestrae increases, making the microvessels the major sites of fenestrae and potential myoendothelial junctions (MEJs). In resistance vessels, fenestrae of the IEL can harbor MEJs – sites of direct physical contact and communication between ECs and VSMCs (Heberlein et al., 2010). In short, MEJs are considered “signaling hubs” between ECs and VSMCs (Straub et al., 2014). Although there is much reported on calcium signaling pathways at the MEJ, there is surprisingly little known about other potential heterocellular communication pathways (Biwer et al., 2016).

The Notch signaling pathway is a highly conserved pathway with four known Notch receptors (Notch 1–4) and several ligands (Jagged 1, 2; delta-like 1, 3, and 4) that are expressed and act in a tissue-specific manner (D’Souza et al., 2010). Activation of the Notch pathway is initiated by a surface ligand on one cell binding and activating a different surface receptor from a neighboring cell (Egan et al., 1998; Artavanis-Tsakonas et al., 1999). This critical pathway directs cell specificity, phenotype, survival, and tissue patterning (Krebs et al., 2000; Uyttendaele et al., 2001; Doi et al., 2006; High et al., 2008; Tang et al., 2010; Boucher et al., 2012; Wang et al., 2012; Lin and Lilly, 2014). In the vasculature, Notch coordinates vasculogenesis through EC recruitment of VSMCs to form intact blood vessels, and more recent data has suggested a role for Notch in the pathophysiology of cancer and diabetes, including data showing that Notch2/Notch3 blockade mitigates tumor growth (Yen et al., 2015). Notch is also important in maintaining the endothelium via Delta-like 4 EC-EC signaling (Rostama et al., 2014), and more recent data suggests that VSMC phenotype can be regulated by heterocellular EC-VSMC Notch signaling (Doi et al., 2006; High et al., 2008; Liu et al., 2010; Tang et al., 2010; Boucher et al., 2012; Wang et al., 2012; Lin and Lilly, 2014). Though its importance in EC-VSMC biology has been demonstrated in developmental models, the specific location of Notch signaling in the intact blood vessel is currently unknown. Most current Notch studies are performed *in vitro*, which eliminates the naturally occurring barrier, the IEL, within the mature blood vessel. Despite a plethora of data regarding the importance of Notch as a critical cell-cell regulator during vascular development, there are currently no known studies that demonstrate that Notch signaling in mature vessels might occur at the MEJ, nor are there any studies to this effect in the intact, *in vivo*, adult coronary circulation. To address these gaps in our knowledge, we tested the hypothesis that Notch expression and signaling occurs at the MEJ located at fenestrae in the IEL. Using a combination of mouse vascular tissue and primary human coronary vascular cells to test this hypothesis, we examined the localization of Notch receptors and ligands at the *in vitro* and *in vivo* MEJ, and we tested Notch signaling activation at *in vitro* MEJs. Understanding the heterocellular underpinnings of Notch signaling in mature, intact blood vessels, particularly within the coronary circulation because its pathophysiology is the leading cause of heart disease, is of absolute importance so that we may better understand and target aberrant Notch signaling in disease.

## Materials and Methods

### Materials and Reagents

All reagents for solutions, unless otherwise specified, were purchased from Fisher Scientific (Waltham, MA, United States). Primary antibodies and stains were as follows: Alexa Fluor 633 Hydrazide (approximates elastin staining), Notch3 (Santa Cruz and Abcam), Jagged1 (Santa Cruz), Notch1 (Abcam), Notch2 (Abcam), and Pai-1 (Abcam). Secondary antibodies were: donkey anti-goat, donkey anti-rabbit or donkey anti-mouse Alexa 488 or Alexa 555, all from Invitrogen. Transwells (polyester, 0.4 μm pore diameter for imaging and 1.0 μm pore for luciferase assay) were purchased from Corning.

#### Animals

Normal male 16 week-old (Db/db; BKS.Cg-*m* + / + Lepr^db^/J) and C57BL/6J mice were obtained from The Jackson Laboratories. They were housed under a 12-h light/dark cycle at 22°C and 60% humidity and were allowed *ad libitum* access to standard low-fat laboratory chow and water. This study was conducted in accordance with the National Institutes of Health Guidelines, and it was approved by the Institutional Animal Care and Use Committee at Nationwide Children’s Hospital.

#### Coronary IEL Immunofluorescence

Paraffin-embedded mouse hearts were sectioned (5–6 μm) for the detection of elastin (Alexa Fluor 633 Hydrazide, Thermo-Fisher), and/or immunohistochemical detection of Notch3, Jagged1, Notch1 and PAI-1. Briefly, sections were deparaffinized, followed by antigen retrieval in a citrate buffer. Sections were blocked in fish skin gelatin and bovine serum albumin. Sections were then incubated overnight in primary antibodies. Slides were then incubated for 1 hr at room temperature with the appropriate secondary antibody and sections were mounted and counter stained using Vectashield with DAPI (Vector Laboratories). Images were taken at 40× magnification on a Zeiss 710 confocal microscope. Figures are representative of composite *z*-stacks. For *z*-stacks, a minimum of 10 stacks at a depth of 0.3 μm/stack were compiled.

### Coronary Notch3 by Immunogold Electron Microscopy

Coronary resistance microvessels (CRMs) from adult C57BL6/J mice were carefully isolated and subjected to processing for immunogold electron microscopy as previously described (Billaud et al., 2011). Briefly, mice were anesthetized using 3% isoflurane, and hearts were perfusion fixed using 4% paraformaldehyde (PFA)/0.5% glutaraldehyde (Glu) and 60 mM KCl until cardiac arrest. CRMs were carefully isolated with surrounding myocardium and fixed in 4% PFA/0.5% Glu for 1 h at 4°C. Tissues were washed and dehydrated in graded steps of ethanol prior to infiltration with LR White resin. 75–80 nm sections were cut onto nickel EM grids, and Notch3 immuno-EM was performed at the MEJ as previously described (Billaud et al., 2011).

#### LAD and Femoral IEL Staining

To verify Notch expression within MEJs of the coronary circulation and other vascular beds, left anterior descending (LAD) coronary arteries and femoral arteries were isolated and immediately placed in physiological saline buffer (PSS, in mM: 130 NaCl, 4 KCl, 1.2 MgSO_4_, 4 NaHCO_3_, 10 HEPES, 1.2 KH_2_PO_4_, 5 glucose, and 2.5 CaCl_2_ at pH 7.4). Arteries were cannulated in a pressure myograph chamber (Living Systems, Inc.) containing PSS and the lumen was perfused and pressurized at 70 mmHg. After a 30 min equilibration period, the vessels were fixed with 4% paraformaldehyde in PSS for 30 min. Next, the vessels were perfused for 10 min, washed ablumenally (3×), and treated with 0.5% Triton X-100 for 20 min. After washing with PSS, lumens were again perfused for 45 min with blocking solution (1% BSA, 0.1% cold water fish skin gelatin, 0.1% Tween 20, and 0.05% NaN_2_ in PBS) while the pressure myograph chamber was filled with the same blocking solution. Primary antibodies were perfused into the lumens for 10 min and the femoral arteries were removed from the cannula and placed in an individual well of a 96 well-plate filled with blocking solution and primary antibody and incubated overnight at 4°C. The next day, the femoral arteries were re-cannulated and perfused with PSS for 10 min. Then, the secondary antibody was perfused lumenally and ablumenally for 45 min. The vessel was then washed both lumenally and ablumenally with PSS to remove excess secondary antibody. To stain elastin, the vessels were ablumenally incubated with Alexa Fluor 633-conjugated sodium hydrazide (Molecular Probes, 0.2 μM in PSS) for 20 min. This technique was based upon previously published methods for IEL elastin visualization (Clifford et al., 2011). The vessels were then washed with PSS via perfusion and three chamber washes. Lastly, the femoral arteries were removed from the cannula at one end, cut longitudinally from the unattached end, and placed on a glass slide with the luminal side facing up and the excess PSS wicked with a Kimwipe. A single drop of hardmount DAPI mounting medium was placed next to the vessel and a coverslip was positioned on the vessel and the mounting medium was allowed to set prior to imaging on a Zeiss 710 confocal microscope.

#### Vascular Cell Co-culture for *in vitro* MEJ Imaging

Vascular cell co-cultures (VCCC) were assembled as previously described (Isakson and Duling, 2005; Biwer et al., 2016, 2017) using human coronary ECs (hcECs) and human coronary VSMCs (hcVSMCs) (Lonza, Morristown, NJ, United States and ATCC, Manassas, VA, United States). In brief, transwell inserts were placed upside down in a large petri dish and coated with fibronectin solution (0.1 mg/mL). Next, approximately 75,000 hcVSMCs were plated onto this side of the transwell for 48 h. After the 48 h, the transwells were flipped and placed into media filled wells in a 6-well dish. Next, the opposite side was coated with a gelatin solution then approximately 360,000 ECs were plated for 48 h. The transwell inserts were fixed in paraformaldehyde after the experiment for imaging. Negative controls were incubated with appropriate secondary antibodies only. Since both Pai-1 and Notch2 required the same secondary antibody, the same negative control was used for those images only. All experiments were performed in at least three replicates or higher.

### Notch Activity at *in vitro* MEJs: VCCC for Luciferase Assay

In separate experiments, prior to co-culture, hcVSMCs (Lonza and ATCC) were transfected with CBF-luciferase (Notch sensor) and CMV-SEAP (transfection control) plasmids. The CBF-luciferase plasmid (pCBF-Luc) contains CBF1-binding elements upstream of the SV40 promoter and was generated as described (Liu et al., 2009) with the pNL1.3 secreted luciferase plasmid (Promega). To measure Notch transcriptional activity, hcVSMCs were placed in a 12-well plate at 6 × 10^4^ cells/well and transfected with plasmids the pCBF-Luc and CMV-SEAP (addgene #24595), as an alkaline phosphatase internal control using Lipofectamine 3000 kit (Invitrogen) for 24 h. The transfected cells were then plated 2.0 × 10^4^ cells/insert on the transwell membrane to create the VCCC as previously described (Isakson and Duling, 2005). After 24 h, the insert was inverted and the upper membrane was plated with hcECs at 8.6 × 10^4^ cells/insert for an additional 72 h in the presence and absence of DAPT (20 μM). Untransfected hcVSMCs, plated across transwells from transfected hcVSMCs, were used as a negative control. The cultured medium was collected and reacted with Nano-Glo (Promega) substrate to measure the secreted luciferase, and Tropix reagent (Applied Biosystems) to measure alkaline phosphatase. Luciferase and phosphatase readings were performed on a LUMIstar Omega luminometer (BMG Labtech). To normalize the transfection efficiency, data were normalized based on an equivalent amount of SEAP activity. All VCCC experiments were performed as *n* = 5 (*n* = 5 different human donors from Lonza and ATCC).

#### Statistical Analysis

All data are expressed as mean ± SEM with a probability of *p* < 0.05 used to denote statistical significance using GraphPad Prism 7.0 (GraphPad Software, La Jolla, CA, United States). A one-way ANOVA followed by a Bonferroni’s *post hoc* test was performed for Notch luciferase activity.

## Results

### Notch Receptors and Ligands Are Expressed Within the IEL Fenestrae and MEJ of Intact Mouse Coronary Resistance Microvessels (CRMs)

**FIGURE 1:**
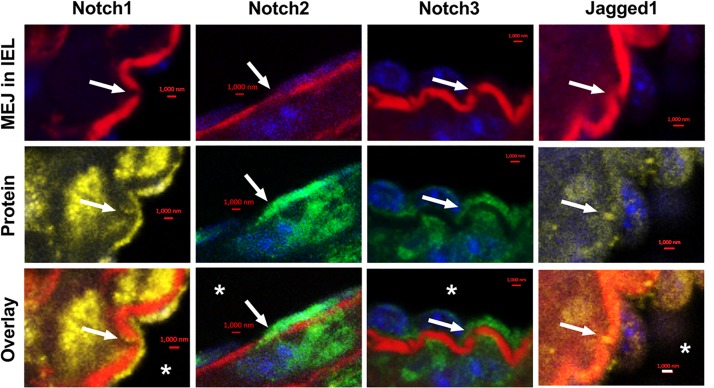
Notch1, Notch2, Notch3, and Jagged1 are expressed at *in vivo* MEJs at IEL fenestrae of intact coronary microvessels. Representative high-magnification images depicting that Notch1, Notch2, Notch3, and Jagged1 are located in the IEL fenestrae (white arrows) of coronary microvessels within the mouse myocardium. * indicates coronary lumen; *n* = 3–5 per group; Scalebars = 1 μm.

**FIGURE 2:**
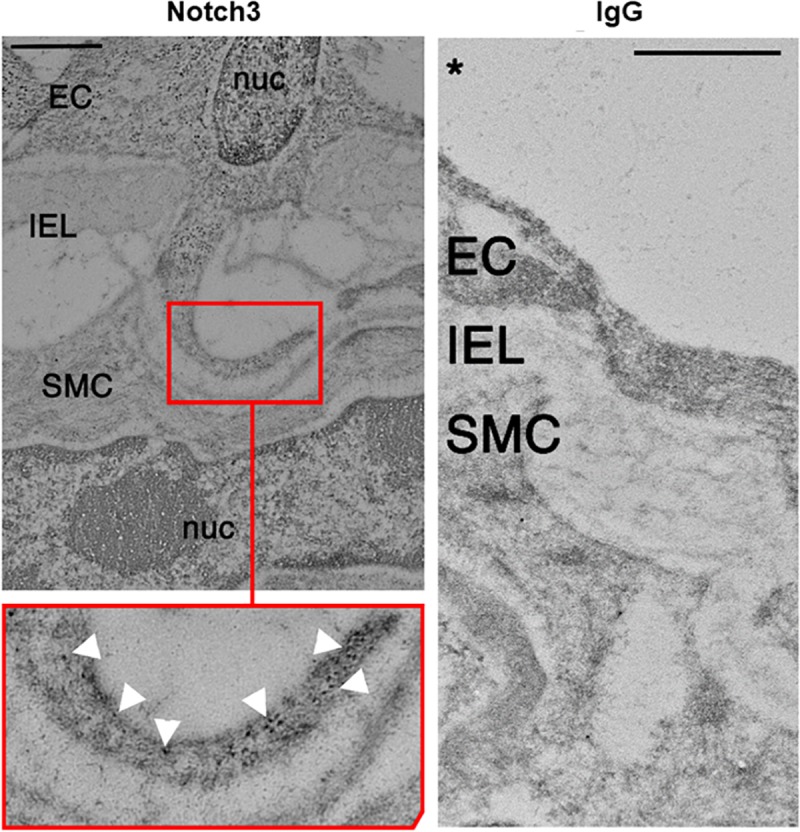
Electron micrograph of Notch3 in the coronary microvascular MEJ. Representative micrograph of Notch3 visualization using immunogold beads, which showed robust expression within the MEJ of normal CRMs highlighted by white arrows in the inset. Note the absence of IgG in the control experiment depicted in the right panel. Scale bar = 0.5 μm.

We examined the localization of Notch receptors and ligands within the fenestrae of intact CRMs IEL. MEJs are recognized as fenestrae, or pores, that are located along the IEL. Utilizing Alexa 633 hydrazide to stain elastin, combined with co-immunostaining for Notch1, Notch2, Notch3, and Jagged1, the fenestrae and protein co-localization within the IEL become apparent. In intact CRMs embedded within heart sections, we observed Notch1, Notch2, Notch3, and Jagged1 expression within the fenestrae of the CRM IEL (Figure 1). Immunostaining clearly shows bridging from the VSMC side of the IEL to the endothelial side in certain IEL fenestrae locations (Figure 1). In normal mice, the frequency of fenestrae in CRM cross-sections was 0.04 ± 0.005 fenestrae/μm of IEL circumference. We confirmed the presence of Notch3 at the CRM MEJ by immunogold EM (Figure 2).

### Notch 2 and Notch3 Proteins Are Located Within MEJs in IEL Fenestrae of Mouse Left Anterior Descending (LAD) Coronary Arteries

**FIGURE 3:**
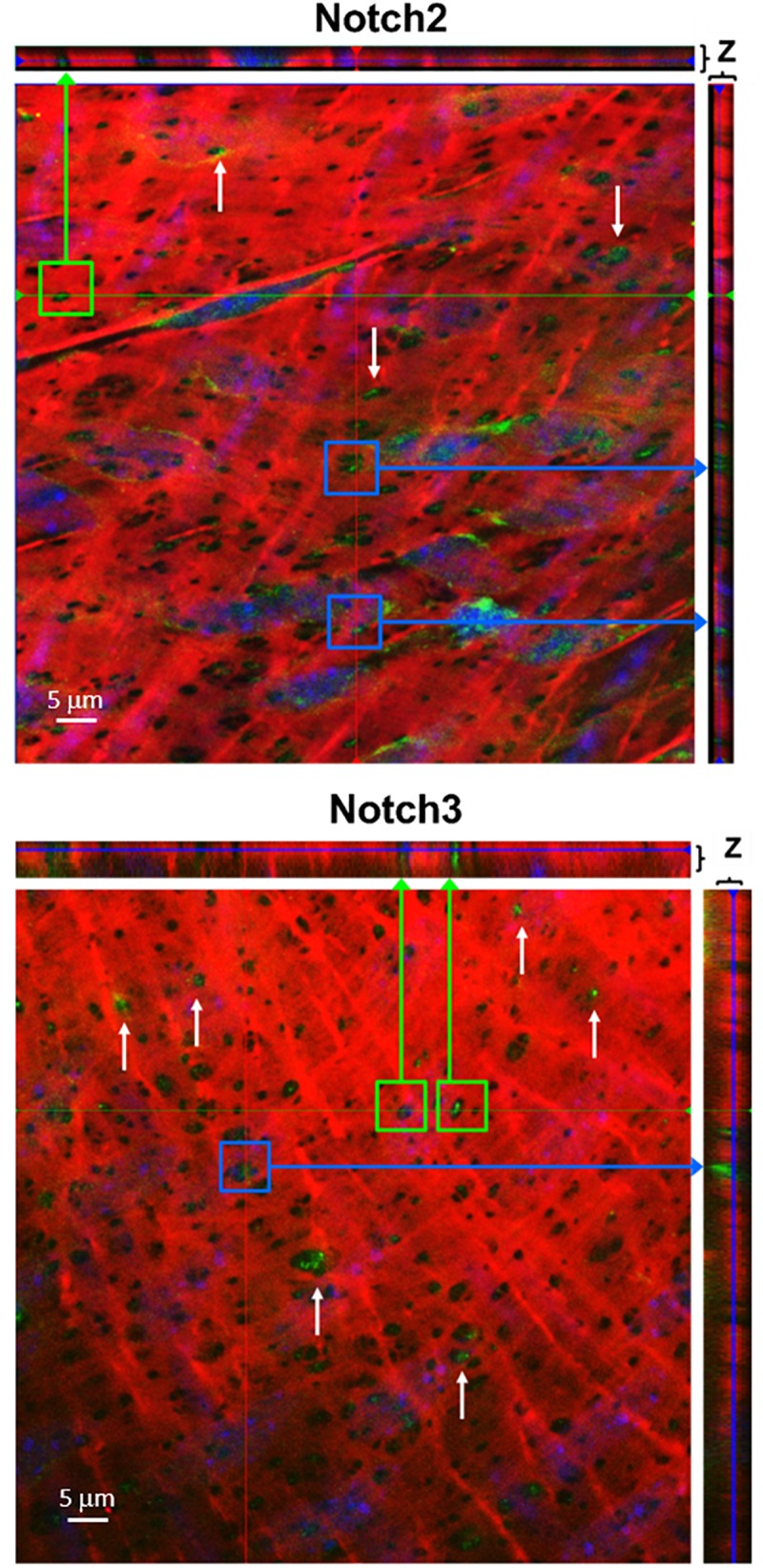
Notch2 and Notch3 are expressed in IEL fenestrae of coronary arteries. Representative confocal images depicting that Notch2 and Notch3 are located in the IEL fenestrae mouse LAD coronary arteries (white arrows). Note that in the *z*-direction of both the *x*- and *y*-axis, the expression of both proteins traverses the entire thickness of the IEL (green and purple arrows, respectively). *n* = 3–5 per group. Scale bar = 5 μm.

To determine whether Notch receptors are located in the IEL fenestrae of larger coronaries, we performed immunofluorescence on mouse LADs using antibodies and Alexa 633 hydrazide. Using confocal microscopy, we observed Notch2 and Notch3 protein immunofluorescence within the punctate holes of the IEL (Figure 3). The *z*-stack representation (top and right of each figure) illustrates that Notch protein expression traverses the entire thickness of the IEL, clearly demonstrating that Notch signaling proteins are expressed within MEJs. We found that, on average, 39% and 33% of IEL fenestrae were positive for Notch2 and Notch3 expression, respectively.

### Notch Receptors and Ligands Are Localized to MEJs in IEL Fenestrae of Mouse Femoral Arteries

**FIGURE 4:**
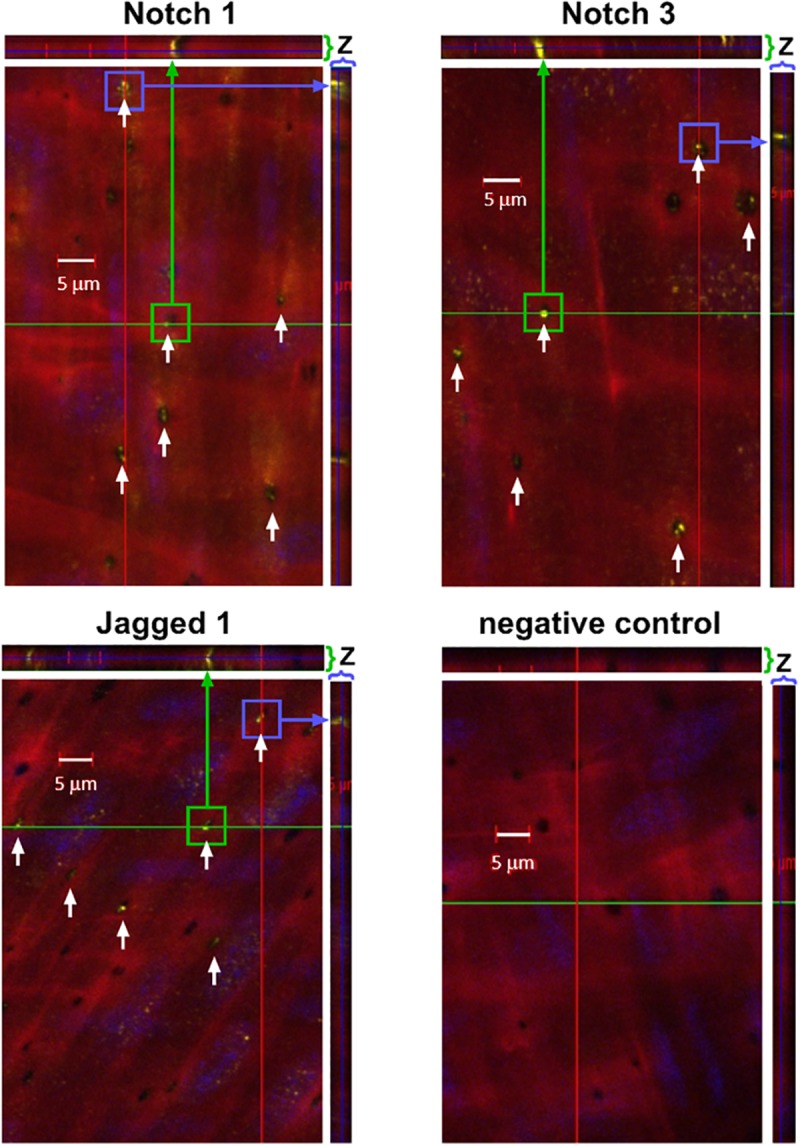
Notch1, Notch3, and Jagged are expressed in IEL fenestrae of femoral arteries. Representative confocal images depicting that Notch1, Notch3, and Jagged1 are located in the IEL fenestrae mouse femoral arteries (white arrows). Note that in the *z*-direction of both the *x*- and *y*-axis, the expression of all three proteins traverses the entire thickness of the IEL (green and purple arrows, respectively). *n* = 3–5 per group; Scale bar = 5 μm.

To determine whether Notch MEJ expression occurs outside of the coronary circulation, we also performed immunofluorescence on mouse femoral arteries using antibodies and Alexa 633 hydrazide. Using confocal microscopy, we observed Notch1, Notch3, and Jagged1 protein immunofluorescence within the punctate holes of the IEL (Figure 4). The *z*-stack representation (top and right of each figure) illustrates that Notch protein expression traverses the entire thickness of the IEL, clearly demonstrating that Notch signaling proteins are localized to MEJs. We found that, on average, 46%, 51%, and 58% of IEL fenestrae were positive for Notch1, Notch3, and Jagged1 expression, respectively.

### Notch Signaling Pathway Is Expressed in the *in vitro* Human Coronary MEJ

**FIGURE 5:**
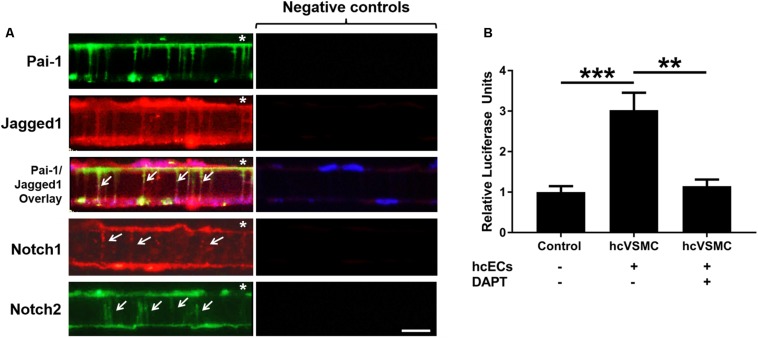
Notch receptors and ligand are located within and active at the cellular extensions of the human *in vitro* MEJ. **(A)** Representative images showing localization of PAI-1, Jagged1, Notch1, and Notch2 in human coronary ECs and human coronary VSCMs cultured on the top and bottom of transwell inserts, respectively. Note that PAI-1 expression is limited to ECs while Jagged1, Notch1, and Notch2 are expressed in both ECs and VSMCs and are also expressed at the MEJs (white arrows). Negative controls were incubated with appropriate secondary antibodies only. Since both Pai-1 and Notch2 required the same secondary antibody, the same negative control was used for those images only. * indicates endothelial side of transwell. *n* = 3–4 per group. Scale bar = 10 μm. **(B)** Human coronary VSMC (hcVSMC) Notch was activated at the *in vitro* MEJ (across transwell inserts) by co-culture with *human* coronary ECs (hcECs). This induction was completely blocked by the γ-secretase inhibitor, DAPT, which prevents Notch signaling activation. *n* = 5 per group. **p < 0.01 and ***p < 0.001.

To determine whether Notch signaling components were localized to the MEJ, we co-cultured primary human coronary ECs (hcECs) and human coronary VSMCs (hcVSMCs) across transwell inserts, and we utilized fluorescent microscopy to immunostain for Pai-1, Jagged1, Notch1, and Notch2 (Figure 5A). Heberlein et al. previously established that PAI-1 is a key factor in the regulation and identification of MEJ formation; therefore, we used PAI-1 as a marker for the visualization of the *in vitro* MEJ (Heberlein et al., 2010). PAI-1 immunofluorescence was observed on the EC side of the transwells and within the transwell pores, indicating the formation of *in vitro* MEJs. We also identified very distinct staining of Notch1, Notch2, and Jagged1 in the pores of the VCCC transwells, which co-localized with PAI-1 within the pores. These data show that Notch signaling components exist within the *in vitro* coronary MEJ.

### Notch Signaling Is Active at the *in vitro* Human Coronary MEJ

Previous data from our group demonstrated an induction of mural cell Notch activity when co-cultured on a monolayer with ECs (Liu et al., 2009; Lin and Lilly, 2014). To assess whether Notch signaling was active at the MEJ, we co-cultured hcECs and hcVSMCs on transwells to create *in vitro* MEJs. Smooth muscle cells were first transfected with a Notch-sensor luciferase plasmid followed by transwell co-culture of the transfected cells with ECs. Notch activity was stimulated by 3-fold across the MEJ in hcVSMC (1.00 ± 0.15 vs. 3.02 ± 0.43, *p* < 0.001), which was completely abrogated by the non-selective Notch γ-secretase inhibitor, DAPT (Figure 5B; 1.15 ± 0.16, *p* < 0.01). These data demonstrate a robust induction of hcVSMC Notch activity by hcECs that occurred at the *in vitro* MEJ, suggesting a role for Notch signaling at these micro-signaling domains in the coronary circulation.

## Discussion

In resistance blood vessels, MEJs are thought to serve as “signaling hubs” between ECs and VSMCs; however, these physical connections are poorly understood, especially in the coronary circulation where tight regulation of blood flow is necessary to maintain proper physiological function. Given that the canonical Notch signaling pathway requires physical cell-cell contact and is critical to vascular development, it would reason that Notch signaling occurs *in vivo* at sites of MEJs, the fenestrae within the IEL and therefore the only space for direct EC-VSMC contact; however, to date, no studies have directly linked Notch signaling to MEJ in mature coronary blood vessels *in vivo*. In this study, we report for the first time that Notch1, Notch2, Notch3, and Jagged1 are expressed within and active at vascular MEJs using both mouse tissues (coronary and femoral) and primary human coronary VSMCs.

Notch signaling between ECs and VSMCs regulate VSMC phenotypic changes and other cellular activities. There are several studies that show Notch signaling activates VSMC differentiation and contractile marker expression (Domenga et al., 2004; Doi et al., 2006; High et al., 2007); however, several studies report that Notch signaling promotes proliferation *in vitro* (Campos et al., 2002; Sweeney et al., 2004; Havrda et al., 2006) and following vascular injury *in vivo* (Wang et al., 2002; Li et al., 2009). Notch2 and Notch3 are the predominant Notch receptors in the vasculature and both have been linked to proliferation in opposite manners (Baeten and Lilly, 2015). Notch1 and Notch4 are the principal Notch receptors in the ECs, which explains why we observed Notch1 in the *in vitro* MEJ (Figure 5). We also observed Notch2 and Notch3 expression in the coronary microvascular MEJ (Figures 1, 2), so their expression is not excluded from ECs in this vascular bed. Data from the Isakson laboratory previously demonstrated that ECs can form the cellular extensions that penetrate the pores of the *in vitro* MEJ (Heberlein et al., 2009), potentially through a plasminogen activator inhibitor-1 (PAI-1) mechanism (Heberlein et al., 2010, 2012). In keeping with this notion, we also observed Jagged1, an endothelial ligand for Notch receptors, Notch1, and Notch2 in the pores of the MEJ (Figure 5A). Importantly, we also found Notch1, Notch3, and Jagged1 all to be localized to the *ex vivo* MEJ in femoral arteries from mice (Figure 4). We next wanted to determine whether Notch signaling components were located with both the microvessels and larger arteries of the coronary circulation. Distinct Notch2 and Notch3 protein expression was clearly visible in the fenestrae of the mouse LADs (Figure 3). We also observed that Notch1, Notch2, Notch3, and Jagged1 all to be located within the *in vivo* MEJ in CRMs from mice (Figure 1). The confocal *z*-stack depicted in Figure 3 nicely demonstrates that the protein expression traverses the entire thickness of the IEL and presented as localized expression within the IEL fenestrae. It is important to note here that there was very little Notch or Jagged immunofluorescence outside of the IEL fenestrae, demonstrating localization of Notch signaling proteins to the MEJ. Finally, using a CBF-luciferase construct, we observed a robust induction of primary human coronary VSMC Notch signaling by primary human coronary ECs that was completely abrogated by DAPT, a drug that prevents Notch signaling activation (Figure 5B). Previous data from the Lilly laboratory demonstrated that ECs can induce a ∼5–10 fold increase in Notch activity in a monolayer co-culture system (Lilly and Kennard, 2009; Liu et al., 2009; Lin and Lilly, 2014). We contend that, by comparison, the 3-fold increase in Notch activity in our study is robust given that EC-VSMC contact in our model was limited to the pores of the *in vitro* MEJ.

Our novel finding that Notch signaling occurs at MEJs in fenestrae of the mouse and human coronary IEL may impart a new research focus with important impact to vascular biology, from both homeostatic and pathophysiological perspectives. Most current Notch studies are performed *in vitro* which eliminates the naturally occurring barrier, the IEL, within the mature blood vessel. In this study, we wanted to address whether Notch signaling components are present in the mature coronary blood vessels and whether these Notch signaling components are located within the IEL fenestrae, holes which allow for EC-VSMC contact. The number and/or size of IEL fenestrae is known to be altered in disease, which has the potential to impact Notch signaling across the MEJ, and therefore alter VSMC behavior. Indeed, several studies demonstrated the presence of fenestrae in the IEL of both mesenteric arterioles (Clifford et al., 2011; Martinez-Lemus et al., 2017) and femoral arteries (Bender et al., 2015), the number of which were altered in western-diet-induced diabetes/obesity. Others have shown that the number of IEL fenestrae is reduced with age and in diabetes in mesenteric arteries (Boerman et al., 2016; Foote et al., 2016). The size of the fenestrae was significantly increased in mesenteric resistance vessels from old rats compared to young rats and significantly decreased in spontaneously hypertensive rats compared to WKY controls (Briones et al., 2003, 2007). While some of these studies speculated that IEL fenestrae dysregulation may represent dysfunction in EC-VSMC nitric oxide signaling, the exact mechanisms remain unclear. Based on our data presented here, we postulate that Notch signaling plays a role in EC-VSMC communication at the MEJ of the intact blood vessel, and together with altered IEL fenestrae in disease, may indicate that it is involved in the pathophysiology of vascular diseases. Collectively, these data show that Notch signaling proteins are located within and convey activity at the MEJ in intact, mature blood vessels. Understanding how these might be dysregulated in disease, particularly in coronary heart disease, may improve our understanding of EC and VSMC homeostasis and phenotype for the direct targeting of the Notch signaling system in coronary heart disease.

### Limitations

The data presented in this study represent an important first step toward understanding the full implications of Notch signaling at the MEJ in mature coronary blood vessels. The purpose of this study was to assess whether Notch receptors and ligands were present and active at the MEJ. While the data show that Notch signaling occurs at the MEJ, whether this is via direct EC-VSMC contact or mediated by some other vesicular trafficking between the two cell types at the MEJ remains to be determined. Based on the function of other proteins found at the MEJ (gap junctions, pannexins, NO signaling) (Straub et al., 2014), we would expect that direct cell-cell contact to be the prevailing mechanism, but further comprehensive studies will be required to determine the exact mode of MEJ communication.

## Data Availability Statement

All datasets generated for this study are included in the article/supplementary material.

## Ethics Statement

The animal study was reviewed and approved by the Institutional Animal Care and Use Committee at Nationwide Children’s Hospital.

## Author Contributions

PM, BI, BL, and AT conceived and designed the experiments. PM, LB, and OC performed the experiments. PM, OC, BL, and AT analyzed the data. All authors contributed to the final manuscript.

## Conflict of Interest

The authors declare that the research was conducted in the absence of any commercial or financial relationships that could be construed as a potential conflict of interest.
